# Early Myocardial Repolarization Heterogeneity Is Detected by Magnetocardiography in Diabetic Patients with Cardiovascular Risk Factors

**DOI:** 10.1371/journal.pone.0133192

**Published:** 2015-07-17

**Authors:** Yi-Cheng Chang, Chau-Chung Wu, Chih-Hung Lin, Yen-Wen Wu, Ying-Chieh Yang, Tien-Jyun Chang, Yi-Der Jiang, Lee-Ming Chuang

**Affiliations:** 1 Graduate Institute of Medical Genomics and Proteomics, National Taiwan University, Taipei, Taiwan; 2 Department of Internal Medicine, National Taiwan University Hospital, HsinChu branch, HsinChu, Taiwan; 3 Department of Internal Medicine, National Taiwan University Hospital, Taipei, Taiwan; 4 Department of Medicine, College of Medicine, National Taiwan University, Taipei; Taiwan; 5 Department of Primary Care Medicine, College of Medicine, National Taiwan University, Taipei, Taiwan; 6 Department of Internal Medicine, Taipei Medical University Hospital, Taipei Taiwan; 7 Department of Nuclear Medicine and^.^ Cardiology Division of Cardiovascular Medical Center, Far Eastern Memorial Hospital, New Taipei City, Taiwan; 8 Departments of Nuclear Medicine, National Taiwan University Hospital, Taipei, Taiwan; 9 National Yang-Ming University School of Medicine, Taipei, Taiwan; 10 Institute of Preventive Medicine, College of Public Health, National Taiwan University, Taipei, Taiwan; Temple University, UNITED STATES

## Abstract

Multi-channel magnetocardiography (MCG) is a sensitive technique to map spatial ventricular repolarization with high resolution and reproducibility. Spatial ventricular repolarization heterogeneity measured by MCG has been shown to accurately detect and localize myocardial ischemia. Here, we explored whether these measurements correlated with cardiovascular risk factors in patients with type 2 diabetes. Two hundreds and seventy-seven type 2 diabetic patients without known coronary artery disease (CAD) and arrhythmia were recruited consecutively from the outpatient clinic of National Taiwan University Hospital. The spatially distributed QTc contour maps were constructed with 64-channel MCG using the superconducting quantum interference device (SQUID) system. Indices of myocardial repolarization heterogeneity including the smoothness index of QTc (SI-QTc) and QTc dispersion were derived and analyzed for association with conventional cardiovascular risk factors. SI-QTc correlated strongly with the QTc dispersion (*r* = 0.70, *p* <0.0001). SI-QTc was significantly higher in patients with presence of metabolic syndrome in comparison to those without metabolic syndrome (8.56 vs. 7.96 ms, *p* = 0.02). In univariate correlation analyses, QTc dispersion was associated with smoking status (average 79.90, 83.83, 86.51, and 86.00 ms for never smokers, ex-smokers, current smokers reporting less than 10 cigarettes daily, and current smoker reporting more than 10 cigarettes daily, respectively, *p* = 0.03), body weight (*r* = 0.15, *p* = 0.01), and hemoglobin A1c (*r* = 0.12, *p* = 0.04). In stepwise multivariate regression analyses, QTc dispersion was associated with smoking (*p* = 0.02), body weight (*p* = 0.04), total cholesterol levels (*p* = 0.05), and possibly estimated glomerular filtration rate (*p* = 0.07). In summary, spatial heterogeneity of myocardial repolarization measured by MCG is positively associated cardiovascular risk factors including adiposity, smoking, and total cholesterol levels.

## Introduction

Myocardial ischemia causes regional ventricular repolarization abnormalities via altered resting membrane potential, shortening of the action potential, and decreased conduction velocity in ischemic zone [1]. The regional electrophysiological difference between normal and ischemic zones generated voltage potentials and electric currents that could be detected by 12-lead body surface electrocardiography (ECG) [1]. Several ventricular repolarization abnormalities recorded by ECG including ST segment elevation/depression, hyperacute T wave, T wave inversion, and QTc dispersion have been widely adopted to detect myocardial ischemia[1].

Magnetocardiography (MCG) measures the magnetic field generated by the same cardiac electric currents without contact of body surface. The signal waveforms measured by 12-lead ECG and MCG were similar but their surface distributions are orthogonal [2]. The recently developed multi-channel MCG technique provides fast three-dimensional mapping of cardiac electric activity with high spatial and temporal resolutions [2]. As compared to 12-lead ECG, MCG is more sensitive to tangential currents, vortex currents, and current flow between endocardium and epicardium with less dependence on body conductance outside the heart [3]. These advantages make MCG an emerging diagnostic tool for myocardial ischemia, especially in patients without significant 12-lead ECG finding. Spatial ventricular repolarization mapping by multi-channel MCG has been shown to accurately detect and localize myocardial ischemia with superior sensitivity and specificity than 12-lead ECG [4–8]. Previous studies consistently showed increased spatiotemporal ventricular repolarization heterogeneity in patients with coronary artery disease (CAD) [4–8].

Metabolic abnormalities such as obesity, hypertension, hyperglycemia, dyslipidemia, and chronic renal disease are known cardiovascular risk factors. Subjects with metabolic syndrome have 2–5 times higher risk of developing CAD [9–11]. The risk is even higher in diabetic patients with the metabolic syndrome than those with type 2 diabetes alone [11] and therefore metabolic syndrome was viewed as a pre-disease status that warrants intervention [10,11]. However, no study to date had evaluated the correlation between these cardiovascular risk factors and spatial ventricular repolarization heterogeneity in subjects without CAD. In this study, we construct the QTc contour map of 278 type 2 diabetic patients without CAD history using 64-channel MCG. Parameters of myocardial repolarization heterogeneity estimated from QTc contour map were derived and analyzed for their association with various cardiovascular risk factors.

## Materials and Methods

### Subjects

Between June to September 2011, two hundred and seventy-eight type 2 diabetic patients were enrolled from the metabolism clinic of National Taiwan University Hospital (NTUH). Exclusion criteria were angina pectoris, myocardial infarction, cardiac arrhythmias, congestive heart failure, myocardial infarction, angina pectoris, or history of percutaneous coronary angiography or coronary bypass surgery. The study protocol was approved by the Institutional Review Board of National Taiwan University Hospital and written informed consent was obtained from each patient.

### Measurements

Blood pressure was measured by trained nurses after rest for 10 minutes. Waist circumferences were measured midway between the lowest rib and the iliac crest to the nearest 0.1 cm. Fasting plasma glucose was measured after overnight fasting. Plasma glucose and serum total cholesterol, high-density lipoprotein cholesterol (HDL-C), low-density lipoprotein cholesterol (LDL-C), triglycerides, creatinine, and uric acid levels were analyzed by an automatic analyzer (Hitachi 7250 special; Hitachi, Tokyo, Japan). Hemoglobin A1c was measured using the Primus CLC330 Glycohemoglobin Analyzer. Post-meal plasma glucose was measured 2 hours from the start of the meal. Estimated glomerular filtration rate (eGFR) was calculated using the Modification of Diet in Renal Disease (MDRD) Study equation as follows: eGFR (mL/min/1.73 m^2^) = 175 × (serum creatinine)^-1.154^ × (age)^-0.203^ × (0.742 if female) [11]. The metabolic syndrome is defined using the International Diabetes Federation (IDF) definition [12]. A person is defined to have the metabolic syndrome defined by IDF if they have: (1) central obesity (defined as waist circumference > 90 cm in males and 80 cm in females) plus any 2 of the following 4 components: (2) triglycerides≥ 150 mg/dL (3) HDL-C < 40 mg/dL in males or < 50 mg/dL in females (4) systolic blood pressure (SBP) ≥ 130 or diastolic blood pressure (DBP) ≥ 85 mm Hg or treatment of previously diagnosed hypertension (5) fasting plasma glucose ≥ 100 mg/dL or previously diagnosed diabetes. The American Heart Association/National Heart, Lung, and Blood Institute (AHA/NHLBI) definition is similar to the IDF definition except that central obesity is a requisite for definition [13]. Instead, subjects with any 3 (or more) of the 5 components is defined as having a metabolic syndrome by AHA/NHBLI criteria. Smoking status was classified as “never smokers”, “ex-smokers”, “current smokers with less than 10 cigarettes daily”, and “current smokers with more than 10 cigarettes daily” and are coded as ordinal variables. Alcohol use was classified as “less than 2–3 times annually”, “2–3 times monthly” “2–3times weekly”, and “more than 3 times weekly” using questionnaires and are coded as ordinal variables. The anonymous data set were deposited as (S1 Dataset).

### MCG

MCG was performed in a magnetically shielded room using the 64-channel superconducting quantum interference device (SQUID) system. The MCG signals were digitally recorded for 100 seconds at a sampling rate of 500 Hz, with the patient in the supine position and the 2-D arrayed sensors positioned close to the left chest wall. The QT interval was defined from the earliest onset of the QRS complex to the terminal portion of the T wave at each position from the time-averaged Bz (magnetic field at axis Z, i.e., perpendicular to the chest wall) curves by using overlapped MCG waveforms from 64 MCG channel. The QTc represented a QT interval corrected for the previous cardiac cycle length according to Bazett’s formula: QTc = QT / square root of R-R interval. The QTc was used for the construction of the QT contour map, with a spatial resolution of 21×21. Two parameters of myocardial repolarization heterogeneity including QTc dispersion and smoothness index of QTc (SI-QTc) were derived from the QT contour map. The QTc dispersion was defined as the difference between the longest and shortest QTc intervals on the QT contour map. SI-QTc was calculated as follows: SI-QTc = (1/S) Σ (k = 1 to S) [(1/*n*) Σ*n* |(QTc)_*k*_−(QTc)_*n*_|], where S is the total number of measured points, Σ (k = 1 to S) indicates the sum of the all *S* measured MCG points. (1/n) Σn|(QTc)_*k*_−(QTc)_n_| is the spatially averaged QTc at a fixed measured position *k*, summed over the total number of nearest neighbor, *n*. SI-QTc is calculated as the mean of these averaged differences of all channels. SI-QTc increase as the difference of QTc between neighborhood sites increases. The details of these parameters were described previously [7,8,14,15]. The SI-QTc reflected regional repolarization abnormality, while the QTc dispersion reflected global repolarization abnormality.

### Statistical analysis

Skewed variables including SI-QTc, body mass index (BMI), blood pressure, serum triglycerides, fasting glucose, HDL-C, and HbA1c levels were logarithmically transformed to approximate normal distribution. Differences between patients with or without metabolic syndrome were compared using the Student’s *t* test. The correlations between two continuous variables were estimated using Pearson’s correlation test and correlations between a continuous variable and an ordinal variable were estimated using Spearman’s correlation test. All metabolic variables were further entered into stepwise selection for multivariate linear regression modeling with a default entry significance level of 0.15 and an exit significance level of 0.15. All statistical analyses were performed using SAS 9.0 and GraphPad Prism 5.0.

## Results

**Fig 1:**
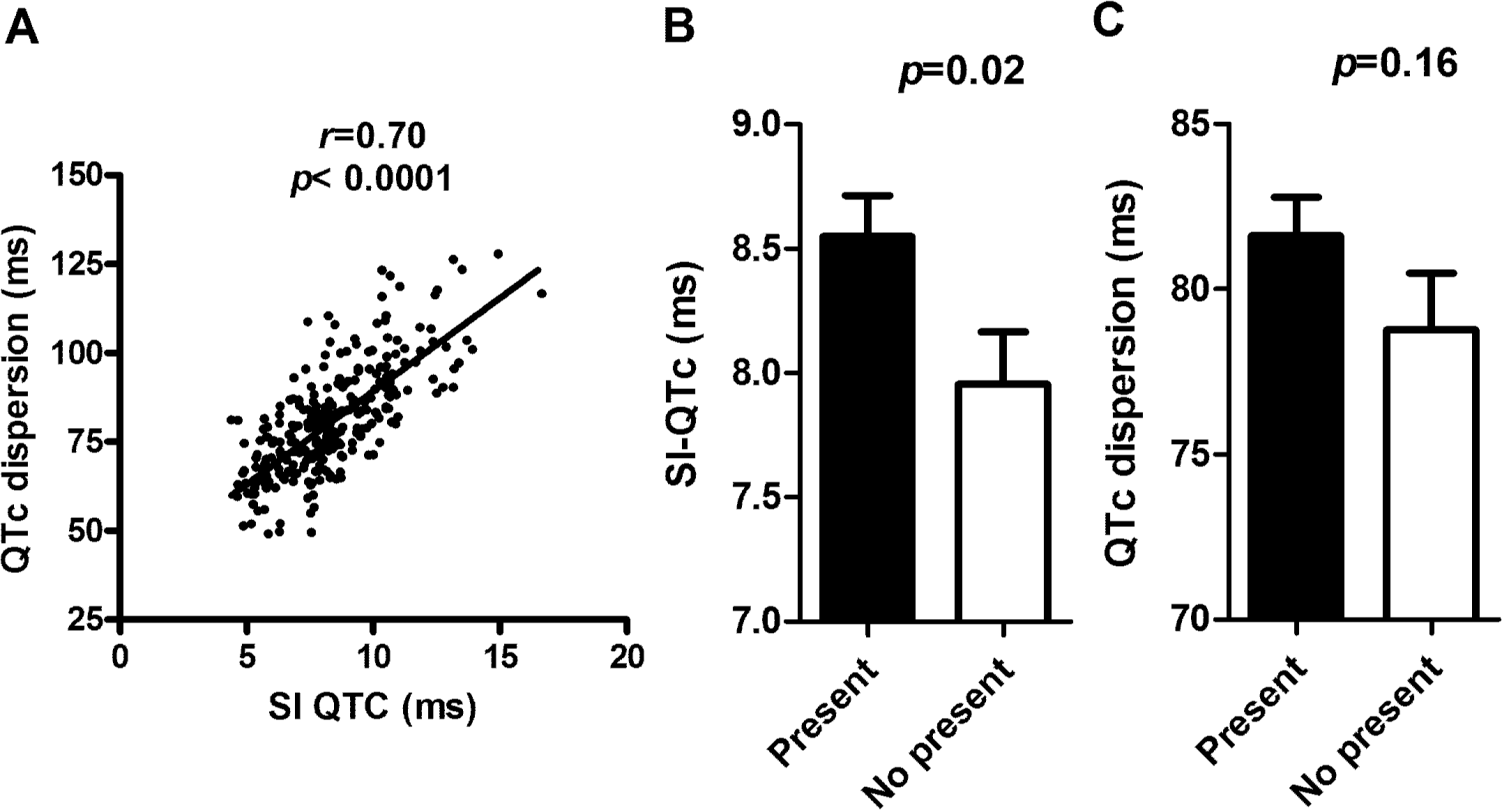
(A) The correlation between QTc dispersion and smoothness index of QTc (SI-QTc). (B) SI-QTc and (C) QTc dispersion in diabetic patients with or without metabolic syndrome.

The baseline characteristics of 278 type 2 diabetic patients were listed in Table 1. SI-QTc correlated well with QTc dispersion (*r* = 0.70, *P*<0.0001, Fig 1A). We classified these patients into those with and without the metabolic syndrome according to the IDF consensus criteria (Table 1). SI-QTc was significantly higher in those with metabolic syndrome in comparison to those without metabolic syndrome (8.57±0.067 vs. 7.99± 0.065 ms, *p* = 0.02, Fig 1B). Similar results were also found using the AHA/NHLBI definition for metabolic syndrome (*p* = 0.02, data not shown). However, QTc dispersion was only non-significantly increased in patients with the metabolic syndrome (81.6±14.8 ms vs. 78.7±15.8 ms, *p* = 0.16, Fig 1C).

**Table 1:** Baseline characteristics of study participants.

Variable	Mean or Count	S.D. or Percentage
N (men/women)	278 (149/129)	53.6 vs. 46.4 (%)
Age (year)	62.40	9.77
Metabolic syndrome (with/without)	164/86	65.6/34.4 (%)
Weight (kg)	66.92	12.63
Body mass index (kg/m^2^)	25.48	4.19
Waist circumference (cm)	91.06	10.90
Systolic blood pressure (mmHg)	134.65	15.82
Diastolic blood pressure (mmHg)	78.82	9.65
Smoking status*	182/30/12/21	74.3/12.2/4.9/8.6 (%)
Alcohol use**	209/23/5/8	85/9.4/2.0/3.3 (%)
Fasting glucose (mg/dL)	131.91	37.04
Post-meal glucose (mg/dL)	175.42	56.21
HbA1c (%)	7.34	1.04
Total cholesterol (mg/dL)	182.52	37.42
Triglycerides (mg/dL)	134.91	106.15
HDL-C (mg/dL)	46.25	13.04
LDL-C (mg/dL)	93.09	29.01
eGFR (ml/min/1.73 m^2^)	72.08	18.40
Sulphonylureas/glinides	211	75.9 (%)
Metformin	235	85.9 (%)
Insulin	51	18.3 (%)
Thiazolidinediones	80	28.8 (%)
Calcium channel blockers	76	27.3 (%)
Beta-adrenergic blockers	33	11.8 (%)
Diuretics	15	5.4 (%)
ARB/ ACE inhibitors	150	54.0 (%)
Statins	115	41.4 (%)
Fibtrates	11	4.0 (%)
SI QTc (ms)	8.22	2.21
QTc dispersion (ms)	80.55	15.16

*Smoking status was classified as “never smokers”, “ex-smokers”, “current smokers with less than 10 cigarettes daily”, and “current smokers with more than 10 cigarettes daily” and are coded as ordinal variables.

**Alcohol use was classified as “less than 2–3 times annually”, “2–3 times monthly” “2–3times weekly”, and “more than 3 times weekly” using questionnaires and are coded as ordinal variables.

SI-QTc, smoothness index of QTc; HbA1c, hemoglobin A1c; HDL-C, high-density lipoprotein-cholesterol; LDL-C, low-density lipoprotein-cholesterol; ACR, albumin-creatinine ratio; eGFR, estimated glomerular filtration rate; ARB: angiogtensin II receptor blocker; ACE, angiotensin converting enzyme

**Fig 2:**
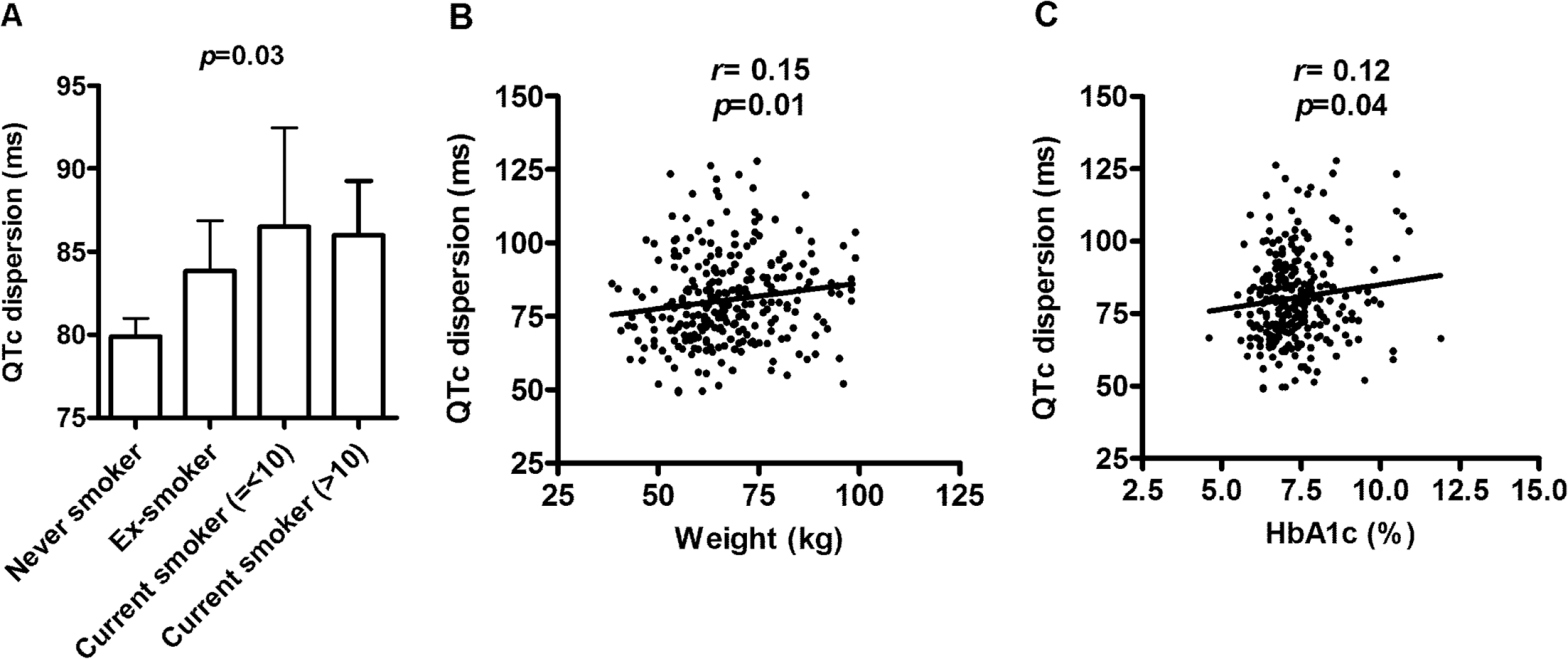
(A) QTc dispersion in never-smoker, ex-smoker, current cigarette smokers reporting < = 10 cigarettes, current smokter reporting more than 10 cigarettes. The correlation between QTc dispersion and (B) body weight and (C) hemoglobin A1c.

We further dissected whether these two parameter are associated with individual cardiovascular risk factor. In univariate correlation analyses, SI-QTc was not associated with any cardiovascular parameter (Table 2). In contrast, QTc dispersion was positively associated with smoking status (*p* = 0.01), body weight (*r* = 0.15, *p* = 0.01), and HbA1c (*r* = 0.12, *p* = 0.04) (Fig 2A–2D). The average QTc dispersion was 79.90, 83.83, 86.51, and 86.00 ms for never smokers, ex-smokers, current smokers reporting less than 10 cigarettes daily, and current smoker reporting more than 10 cigarettes daily (*p* = 0.03) (Fig 2A). QTc dispersion was also borderline associated with other measures of adiposity including BMI (*r* = 0.11, *p* = 0.07) and waist circumference (*r* = 0.11, *p* = 0.07) (Table 2). Neither parameter are associated with medications that have been reported to interfere with ventricular repolarization including sulphonylureas/glinides, calcium channel blockers, beta-adrenergic blockers, diuretics, statins, or angiotensin II receptor blockers/angiotensin-converting enzyme inhibitors (data not shown).

**Table 2:** Correlation between smoothness index of QTc (SI-QTc) and QTc dispersion and metabolic parameters.

Variable	SI-QTc		QTc dispersion	
	Correlationcoefficients	*P*-value	Correlation coefficients	*P*-value
Age	0.0005	0.95	-0.07	0.19
Smoking status*	0.02	0.73	0.14	**0.03**
Alcohol use**	0.03	0.62	0.07	0.28
Weight (kg)	0.071	0.24	0.15	**0.01**
Body mass index (kg/m^2^)	0.085	0.16	0.11	0.07
Waist circumference (cm)	0.079	0.19	0.11	0.07
Systolic blood pressure (mmHg)	0.032	0.59	0.040	0.50
Diastolic blood pressure (mmHg)	0.069	0.25	0.060	0.32
Fasting glucose (mg/dL)	-0.025	0.68	0.055	0.36
Post-prandial glucose (mg/dL)	-0.024	0.69	0.036	0.55
HbA1c (%)	0.055	0.36	0.12	**0.04**
Total cholesterol (mg/dL)	0.023	0.69	0.094	0.12
Triglycerides (mg/dL)	0.054	0.36	0.088	0.14
HDL-C (mg/dL)	-0.041	0.49	-0.026	0.66
LDL-C (mg/dL)	-0.039	0.52	0.004	0.94
eGFR (ml/min/1.73 m^2^)	-0.005	0.92	0.07	0.24

*Smoking status was classified as “never smokers”, “ex-smokers”, “current smokers with less than 10 cigarettes daily”, and “current smokers with more than 10 cigarettes daily” and are coded as ordinal variables.

**Alcohol use was classified as “less than 2–3 times annually”, “2–3 times monthly” “2–3times weekly”, and “more than 3 times weekly” using questionnaires and are coded as ordinal variables.

HbA1c, hemoglobin A1c; HDL-C, high-density lipoprotein-cholesterol; LDL-C, low-density lipoprotein-cholesterol; eGFR, estimated glomerular filtration rate

We performed multivariable regression analyses to identify those independently associated with SI-QTc and QTc dispersion by entering all cardiovascular parameters into stepwise selection. The SI-QTc was only borderline correlated with BMI (*p* = 0.15) (Table 3), while QTc dispersion was positively correlated with smoking status (*p* = 0.02), body weight (*p* = 0.04), total cholesterol levels (*p* = 0.05), and, to a less extent, eGFR (*p* = 0.07) (Table 4).

**Table 3:** Stepwise multiple regression for determinants of smoothness index of QTc (SI-QTc) in type 2 diabetic patients.

Step	Independent variables	Beta (SE)	*P*-value
1	Body mass index (log kg/m^2^)	0.133 (0.094)	0.15

*Multiple linear regressions using stepwise selection was performed with a default entry significance level of 0.15 and an exit significance level of 0.15.

**Table 4:** Stepwise multiple regression for determinants of QTc dispersion in type 2 diabetic patients.

Step	Independent variables	Beta (SE)	*P*-value
1	Smoking status	2.38 (1.04)	**0.02**
2	Body Weight (kg)	0.15 (0.0076)	**0.04**
3	Total cholesterol (mg/dL)	0.051 (0.026)	0.05
4	eGFR (ml/min/1.73 m^2^)	0.093 (0.050)	0.07

*Multiple linear regressions using stepwise selection was performed with a default entry significance level of 0.15 and an exit significance level of 0.15. eGFR, estimated glomerular filtration rate

## Discussion

In this present study, we identified independent associations of QTc dispersion with cardiovascular risk factors including smoking status, body weight, and total cholesterol levels in 278 diabetic patients without known CAD. Another parameter of spatial ventricular repolarization heterogeneity, SI-QTc was significantly increased in diabetic patients with metabolic syndrome as compared to those with diabetes alone. These data indicate regional ventricular repolarization abnormality may develop early in the presence of cardiovascular risk factors and/or metabolic syndrome in type 2 diabetic patients without overt CAD. To our knowledge, this is the first study to investigate the association between spatial ventricular repolarization heterogeneity and cardiovascular risk factors using MCG in human.

Our findings were consistent with previous studies showing that smoking increased ventricular repolarization heterogeneity measured by 12-lead ECG. In a study involving 1,394 young healthy men, smoking was associated significantly lower QT maximum, lower QT minimum, increased spatial QRS-T angle, and spatial T amplitude as compared to non-smokers [16]. Although our studies excluded patients with previous history of CAD, it is possible that smoking still cause clinically silent coronary arterial occlusion, leading to myocardial ischemia and repolarization abnormality. Alternatively, smoking may directly modulate myocardial membrane repolarization. For example, in a study of 30 young men, cigarette smoking acutely increased maximal QT interval and QT dispersion measured by 12-lead ECG [17]. Another study in healthy individuals and 20 CAD patients revealed that QTc increased significantly only during smoking [18].

We also found cholesterol levels positively correlated with QTc dispersion measured by MCG. Consistently, a delicate study demonstrated that rabbits fed with high-cholesterol (HC) diet for 12 week developed longer QT interval, greater QTc dispersion, longer action potential, and increased heterogeneity of repolarization measured by 12-lead ECG with higher peak inward calcium current (*I*ca) [19]. In another study using MCG, induced T-wave abnormality was detected in rabbits fed with HC diet for only 3 weeks [20]. It is worthy to note that conventional 12-lead ECG cannot distinguish the repolarization difference between normal and HC-fed rabbit until having been fed with HC diet for 12 weeks [19,20]. Therefore, these data indicated MCG may be a more sensitive tool than body surface ECG to detect early ventricular repolarization abnormalities induced by metabolic changes.

In our study, QTc dispersion is independently associated with body weight and SI-QTc is borderline associated with BMI. These data are in part in line with previous studies using 12-lead ECG. In a study involving 67 women, significant higher QTc dispersion was found in obese women as compared to non-obese women (57± 23 vs. 38± 15 ms, *p*<0.001) [21]. In another study involving 36 subjects, maximum QTc, minimum QTc, and QTc dispersion measured by 12-lead ECG were greater in obese subjects (57±19 ms) than in the control group (32±13 ms, *p* < 0.0001) [22]. In another study of 63 morbidly obese subjects on liquid protein diet, QT dispersion measured by 12-lead ECG shortens after weight loss [23]. However, another study comparing 54 uncomplicated (i.e., without heart disease, hypertension, diabetes, or other major systemic disease) obese, 35 overweight, and 57 normal weight healthy controls found no difference of QTc dispersion among the three groups (56.4± 2.6 ms, 56.7± 2.1 ms, and 59.4± 2.1 ms) [24]. The inconsistency between studies may result from small sample sizes and difference in study design. Since body surface ECG recoding is influenced by the body composition and conductivity of tissue between electrodes and heart, MCG may be a better tool to analyze the association between adiposity and spatial ventricular repolarization heterogeneity. Taken together, modest evidence support increased heterogeneity of ventricular depolarization in obese subjects.

Metabolic syndrome is a stronger CAD risk factor in diabetic patients than in general population [11]. Indeed, we found that SI-QTc was significantly increased in diabetic patients with metabolic syndrome, although QTc dispersion was only marginally increased in those with the metabolic syndrome. The reason underlying the discrepant associations of these two parameters with metabolic syndrome is currently unknown. But, it should be noted that SI-QTc is a measure of regional smoothness of the QTc contour mapping. In contrast, QTc dispersion, defined as the difference between the longest and shortest QTc on the QTc contour map, is a more global index of repolarization heterogeneity. Consistent with our findings, a previous study involving 83 subjects also found subjects with uncomplicated metabolic syndrome have a greater dispersion of ventricular repolarization time measured by 12-lead ECG [25].

The mechanism by which these metabolic disturbances are related to ventricular repolarization abnormalities remained to be investigated. These metabolic risk factors may either cause coronary arterial occlusion, leading to myocardial repolarization abnormality or directly influence myocardial membrane repolarization. Obesity is associated with elevated sympathetic tone which may influence membrane potentials [26] and is characterized by elevated fatty acids, which has been shown to prolong repolarization [27]. Both obesity and smoking are associated with increased oxidative stress, which could also alter mitochondrial membrane potential and intracellular calcium homeostasis [28, 29]. Elevated cholesterol has been demonstrated to stimulate sympathetic nerve sprouting and electric remodeling [19]. The incorporation of cholesterol into cardiac sarcrolemmal vesicles could also stimulate Na^+^ and Ca^2+^ exchange activity [30].

The major strength of this study is the utilization of multi-channel MCG for evaluation ventricular repolarization heterogeneity. QT dispersion measured by 12-lead ECG, defined simply as the difference between the longest and the shortest QT within 12-leads, was originally proposed as a measure of spatial dispersion of ventricular repolarization. Abundant studies demonstrated that QT dispersion measured by 12-lead ECG is increased in patients with myocardial ischemia, cardiac hypertrophy and dilatation, and QT prolong syndrome [31–35]. However, subsequent studies did not yield similar results [36] and substantial concerns arise about the methodology [37–39]. Later, it was shown that QT dispersion measure by 12-lead ECG does not directly reflect the heterogeneity of recovery times and that it results mainly from variations in the T loop morphology [37–39]. QT dispersion by ECG is a simplified and approximate measure of ventricular repolarization heterogeneity [35]. In contrast to 12-lead ECG, the multi-channel MCG provides high-resolution three-dimensional QT contour mapping that are able to localize regional repolarization abnormality. MCG have also been shown to be more sensitive to tangential currents than 12-lead ECG and can detect circular vortex currents that are not detected by 12-lead ECG [40–43]. Our finding provided further evidence that MCG may detect early myocardial repolarization abnormality even in pre-disease status (i.e. high-risk patients without clinically evident CAD). Spatial repolarization heterogeneity measured by MCG was shown to predict ventricular arrhythmia and sudden cardiac death in patients after myocardial infarction, patients with Brugada syndrome or beta-thalassemia major [14, 43–45]. Therefore, spatial repolarization heterogeneity detected by MCG may be a new predictive tool for ventricular arrhythmia and sudden cardiac death in these high-risk patients. However, the clinical application of MCG is currently limited because of the cost and the set-up requirement. Due to the lack of standardization, the definite sensitivity and specificity of MCG to detect arrhythmogenic risk or myocardial ischemia is still under debate [46]. For detection of myocadial ischemia, most research reported the sensitivity and specificity of rest MCG are both above 70–75% [46].

On the other hand, there are several limitations of this study. First, we did not have coronary angiographic data for all patients. Since diabetes mellitus have been viewed as a cardiovascular disease equivalent, we could not exclude the possibility that some patients may have occult CAD. However, our study design may actually represent real-world clinical scenarios. Second, given the multiple cardiovascular parameters tested, falsely positive association could not be excluded. Third, since these metabolic parameters are closely related, it is still difficult to dissect the complex causal relationship between these parameters and SI-QTc/ QTc dispersion despite the use of multivariate regression. We also did not perform ECG simultaneously in these patients. Therefore, direct comparison of ECG and MCG parameters is not feasible.

## Conclusion

In conclusion, using high-resolution spatial QT contour mapping by MCG, we identified that cardiovascular risk factors including adiposity, smoking, and cholesterol levels are associated with regional ventricular repolarization heterogeneity in diabetic patients without overt CAD.

## Supporting Information

S1 Dataset Delinked dataset of patients. (XLSX)
